# Mesenchymal stem cell‐based treatments for stroke, neural trauma, and heat stroke

**DOI:** 10.1002/brb3.526

**Published:** 2016-08-03

**Authors:** Yogi Chang‐Yo Hsuan, Cheng‐Hsien Lin, Ching‐Ping Chang, Mao‐Tsun Lin

**Affiliations:** ^1^ Meridigen Biotech CO., Ltd.NeihuTaipeiTaiwan; ^2^ Department of Medical ResearchChi Mei Medical CenterTainanTaiwan

**Keywords:** heatstroke, Ischemic stroke, mesenchymal stem cells, neural trauma

## Abstract

**Background:**

Mesenchymal stem cell (MSC) transplantation has been reported to improve neurological function following neural injury. Many physiological and molecular mechanisms involving MSC therapy‐related neuroprotection have been identified.

**Methods:**

A review is presented of articles that pertain to MSC therapy and diverse brain injuries including stroke, neural trauma, and heat stroke, which were identified using an electronic search (e.g., PubMed), emphasize mechanisms of MSC therapy‐related neuroprotection. We aim to discuss neuroprotective mechanisms that underlie the beneficial effects of MSCs in treating stroke, neural trauma, and heatstroke.

**Results:**

MSC therapy is promising as a means of augmenting brain repair. Cell incorporation into the injured tissue is not a prerequisite for the beneficial effects exerted by MSCs. Paracrine signaling is believed to be the most important mediator of MSC therapy in brain injury. The multiple mechanisms of action of MSCs include enhanced angiogenesis and neurogenesis, immunomodulation, and anti‐inflammatory effects. Microglia are the first source of the inflammatory cascade during brain injury. Cytokines, including tumor necrosis factor‐α, interleukin‐1β, and interleukin‐6, are significantly produced by microglia in the brain after experimental brain injury. The proinflammatory M1 phenotype of microglia is associated with tissue destruction, whereas the anti‐inflammatory M2 phenotype of microglia facilitates repair and regeneration. MSC therapy may improve outcomes of ischemic stroke, neural trauma, and heatstroke by inhibiting the activity of M1 phenotype of microglia but augmenting the activity of M2 phenotype of microglia.

**Conclusion:**

This review offers a testable platform for targeting microglial‐mediated cytokines in clinical trials based upon the rational design of MSC therapy in the future. MSCs that are derived from the placenta provide a great choice for stem cell therapy. Although targeting the microglial activation is an important approach to reduce the burden of the injury, it is not the only one. This review focuses on this specific aspect.

## 1 Introduction

### 1.1 Neuroinflammation is a hallmark of brain injury

Inflammation is a hallmark of stroke (Lambertsen, Biber, & Finsen, 2012), traumatic brain injury (TBI) (Mannix & Whalen, 2012), and heatstroke pathology (Chen, Lin, & Chang, 2013). The cytokines that modulate tissue injury in ischemic stroke, TBI, spinal cord injury (SCI), or heatstroke, including tumor necrosis factor‐α (TNF‐α), interleukin (IL)‐1, and IL‐6, are potential targets for future therapy. The production of these cytokines in greatly increased by microglia in the brain the first 24 hours after experimental stroke (Clausen, Lambertsen, Meldgaard, & Finsen, 2005; Clausen et al., 2008; Hill et al., 1999; Lambertsen, Meldgaard, Ladeby, & Finsen, 2005). Interleukin‐1β and TNF‐α are produced by a largely segregated population of microglia and infiltrating macrophages after ischemic stroke in mice (Clausen et al., 2008). This has promoted the hypothesis that inhibiting proinflammatory cytokine production may be a therapeutic approach in treating brain injury (Barone & Parsons, 2000). Indeed, according to an observational study that involved 629 consecutive patients with chronic neurological, neuropsychiatric, and clinical impairment after stroke and TBI, the perispinal administration of etanercept produces clinical improvement (Tobinick, Rodriguez‐Romancce, Levine, Ignatowski, & Spengler, 2014). In addition, various drugs or strategies improve outcomes of experimental heatstroke by reducing the overproduction of these proinflammatory cytokines resulting from heat stress (Chen et al., 2013).

### 1.2 Microglial activation is involved in brain injury pathology

In contrast to their well‐known deleterious roles, TNF‐α and IL‐6 have also been shown to exhibit neuroprotective properties. In both TNF‐deficient mice (Bruce et al., 1996; Gary, Bruce‐Keller, Kindy, & Mattson, 1998; Lambertsen et al., 2009; Taoufik et al., 2007) and IL‐6‐deficient mice (Herrmann et al., 2003), infarct sizes were significantly increased following cerebral injury. In addition, TNF‐α and IL‐6 double‐receptor knockout mice had higher mortality rates than did their wild‐type controls following heatstroke collapse (Leon, Blaha, & DuBose, 2006). Adult IL‐6 knockout mice have also shown to compromise neurogenesis (Bowen, Dempsey, & Vemuganti, 2011). A complete lack of TNF‐α or IL‐6 might be detrimental to neurogenesis in the adult brain (Monje, Toda, & Palmer, 2003; Vallières, Campbell, Gage, & Sawchenko, 2002). This can be concluded by previous studies that show that an appropriate baseline level of TNF‐α or IL‐6 is necessary and essential for neurogenesis or host defense, whereas higher levels of TNF‐α or IL‐6 are detrimental to neurogenesis or host defense.

Microglia are activated rapidly in response to central nervous system injury and produce proinflammatory cytokines, growth factors, reactive oxygen species, nitric oxide, and glutamate (Block & Hong, 2005; Jin, Yang, & Li, 2010; Stolp & Dziegielewska, 2009). An appropriate state of microglial activation is necessary and crucial for host normal neurogenesis and defense; however, microglial overactivation results in deleterious and neurotoxic consequences. Proinflammatory cytokines, such as TNF‐α, IL‐1β, and IL‐6, which are increasingly expressed during experimental stroke, have a crucial role in the progression of neuronal loss and brain injury (Banati, Gehrmann, Schubert, & Kreutzberg, 1993; Barone et al., 1997; Rothwell, Allan, & Toulmond, 1997).

Recent developments in magnetic resonance (MR) and positron emission tomography (PET) imaging techniques have demonstrated that increased binding of the peripheral benzodiazepine receptor (PBR) PET ligand ^11^C‐RK11195 is interpreted as a marker of microglial activation and hence neuroinflammation in several brain diseases (Denes et al., 2010). Increases in ^11^C‐PK11195 binding are found 30 days after stroke in patients, which suggests a contribution of microglial activation to ongoing processes in the ischemic brain (Price et al., 2006). In a rat stroke model, evidence supports a role for microglia as a central mediator in the ongoing processes of stroke damage (Gelosa et al., 2014). In addition, microglial activation is involved in other neurodegenerative disease models, such as Alzheimer's and Parkinson's disease (Mosher & Wyss‐Coray, 2014; Walker et al., 2014), traumatic brain injury (Chio, Lin, & Chang, 2015), and heatstroke (Chen et al., 2013).

Tumor necrosis factor‐alpha levels in both serum and cerebrospinal fluid are found to be significantly elevated in ischemic stroke, traumatic brain injury, and heatstroke (Chen et al., 2013; Chio et al., 2015; Gelosa et al., 2014). Activation of TNF receptor 1 (TNF‐R1) is believed to promote proinflammatory and proapoptotic action, astrogliosis, leukocyte extravasation, and disrupted blood–brain barrier (BBB) permeability (McCoy & Tansey, 2008). However, other results have demonstrated that TNF‐R1 is required for erythropoietin receptor and vasculoendothelial growth factor expression and protective effects in primary cortical neurons after ischemic and excitotoxic injury (Taoufik et al., 2008).

### 1.3 MSC therapy may improve outcomes of brain injury by modulating microglial activation

Mesenchymal stem cells (MSCs) can be derived from different sources, including bone marrow, adipose tissue, the umbilical cord, and the placenta. Currently, clinical trials are being conducted to investigate the therapeutic effects of human MSCs in many cardiovascular and neurodegenerative disorders (Kalladka & Muir, 2014; Mastri, Lin, & Lee, 2014). In addition to their multilineage differentiation potential, MSCs may exert their regenerative effect via the production of multiple paracrine factors (Kalladka & Muir, 2014; Mastri et al., 2014). Production of IL‐6, vascular endothelial growth factor (VEGF), hepatocytes growth factor (HGF), brain‐derived neurotrophic factors (BDNF), glial‐derived neurotrophic factor (GDNF), neurotrophin‐3 (NT3), fibroblast growth factor (FGF), and thrombospondins can be promoted by MSCs. It is well known that neural injury results in BBB breakdown and the infiltration of tissue neutrophils and macrophages into damaged brain tissue, which causes microglial activation. In addition, microglia have been promoted as a compelling target for treating infectious and inflammatory diseases of the brain (Chio et al., 2015; Denes et al., 2010; Rock & Peterson, 2006). It is likely that MSC therapy may improve outcomes of brain injury by modulating microglial activation. Although targeting the microglial activation is an important approach to reduce the burden of the injury, it is not the only one.

### 1.4 MSCs fulfill the criteria that have been established by the international society of cellular therapy

Mesenchymal stem cells are multipotent, self‐renewing cells (Friedenstein, Petrakova, Kurolesova, & Frolova, 1968). They fulfill the following criteria that have been established by the International Society of Cellular Therapy (Dominici et al., 2006): (i) adherence to plastic, (ii) expression of CD105, CD73, and CD90; lack of expression of CD45, CD34, CD14, CD116, CD79a, CD19, and HLA11; and (iii) ability to differentiate into osteoblasts, adipocytes, and chondroblasts in vitro. Due to extensive self‐renewal capacity, their ease of isolation, and their presence during young and fetal life, MSCs that are derived from the placenta are an appropriate source for stem cell therapy.

In this review, we collected publications that pertain to MSC therapy and cerebral injury that is caused by stroke, neural trauma, and heatstroke. In doing so, we emphasized the mechanisms of MSC therapy‐related neuroprotection, which were identified using an electronic search (e.g., using PubMed). It reports the feasibility of MSCs to improve neurological function after injury. It focuses on adult injuries such stroke, TBI, and heatstroke. It summarizes the pathophysiology of the injury briefly and offers an overview of MSCs therapeutic approaches.

## 2 Therapeutic Effects of MSCs in Ischemic Stroke

### 2.1 Neonatal stroke rats or mice

Neonatal stroke occurs frequently in live birth and presents motor dysfunction, cognitive deficits, and epilepsy (Ferriero, 2004; Kirton & de Veber, 2009). However, treatment options are not currently available. The transplantation of MSCs into neonatal animal models of ischemic stroke promotes functional recovery by stimulating neurogenesis, oligodendrogenesis, and axonal remodeling (van Velthoven, Kavelaars, van Bel, & Feijene, 2010a,b; Yasuhara et al., 2008). The beneficial effect of MSC transplantation might involve the augmentation of the secretion of growth and differentiation factors and the fostering of an environment that stimulates both angiogenesis and neurogenesis (van Velthoven et al., 2010b, 2012, 2013) (Table 1). The secretome that has been obtained from MSCs contains several neurotrophic factors, including insulin‐like growth factor‐1 and brain‐derived neurotrophic factor, which are responsible for the protective effects of MSCs that were observed in studies with in vitro and in vivo neuronal injury models (Wei et al., 2009). When compared with adults, it is believed that newborns benefit more from cell therapy because newborns have an increased brain plasticity as well as a different pathophysiology of the injury. In addition, in newborns the microglial activation is more pronounced as microglial activation is present during physiological brain development as well.

**Table 1:** Effects of mesenchymal stem cells (MSCs) therapy on ischemic stroke damage

Treatment regimens	Main results	References no.
1. Neonatal stroke rats or mice received intranasal or intracerebral injection of MSCs	Decreasing cerebral damage by reducing both overproduction of IL‐6 and TNF‐α and microgliosis, but stimulating neurogenesis (e.g., increased production of HGF, VEGF, IGF, EGF, 6FGF, IL‐10, GDNF, BDNF, NF3, angiopoietin, TGF, and I‐CAM 1	van Velthoven et al. (2010a,b), Yasuhara et al. (2008), van Velthoven et al. (2012, 2013), Wei et al. (2009)
2. Adult stroke rats received intravenous or intracerebral injection of MSCs	Decreasing cerebral damage by stimulating synaptogenesis and vessel density, reducing apoptosis in the ischemic boundary zone, and increasing proliferation of progenitor cells in the subventricular zone.	Wakabayashi et al. (2010), Xu et al. (2010); Bao et al. (2011), Lin et al. (2011); Walker et al. (2010), Wei et al. (2012), Ma et al. (2013), Tang et al. (2014a,b), Cheng et al. (2015)
3. Adult stroke monkeys received intracerebral injection of MSCs	Reducing cerebral damage by stimulating production of IL‐10	Li et al. (2010)
4. Adult stroke patients received intravenous injection of MSCs	Reducing cerebral damage by promoting nerve cell proliferation	Weimann et al. (2003) Bang et al. (2005) Lee et al. (2010)

MSCs, mesenchymal stem cells; IL‐6, interleukin‐6; TNF‐α, tumor necrosis factor‐α; IL‐10, interleukin‐10; VEGF, vascular endothelial growth factor; HGF, hepatocytes growth factor; BDNF, brain‐derived neurotrophic factor; GDNF, glial‐derived neurotrophic factor; NT3, neurotrophin‐3; FGF, fibroblast growth factor; IGF‐1, insulin‐like growth factor; EGF, epidermal growth factor; TGF, transforming growth factor; ICAM‐1, intercellular adhesion molecule‐1.

### 2.2 Adult ischemic stroke models

#### 2.2.1 MSC therapy improves outcomes of stroke mainly by secreting paracrine factors

Mesenchymal stem cells have the potential to differentiate into osteoblasts, chondrocytes, adipocytes, hepatocytes, and neurons (Sanchez‐Ramos et al., 2000). Although, MSCs are able to pass through the BBB (Kopen, Prockop, & Phinney, 1999), MSCs that are transplanted by intracerebral or intravenous routes minimally and selectively migrate to the ischemic boundary sites (Li et al., 2002; Zhao et al., 2002). Considering the small number of MSCs in injured brain tissue, the presence of therapeutic neurotrophic factors that are secreted by MSCs apparently confers neuroprotection. This suggests that providing the therapeutic molecules that are secreted by these cells can be neuroprotective (Borlongan, Hadman, Sanberg, & Sanberg, 2004). Although MSCs have been shown to localize only to the injured brain using immunohistochemistry (Chen et al., 2001; Vendrame et al., 2004), intravenously transplanted human MSCs are functionally involved in repair in ischemic stroke rats, possibly by providing human insulin‐like growth factor 1 (IGF‐1), vascular endothelial growth factor (VEGF), epidermal growth factor (EGF), basic fibroblast growth factor (FGF), and neurotrophic neurotrophic factors to the host brain (Wakabayashi et al., 2010). Xu and colleagues (Xu et al., 2010) further suggested that the transplantation of neuronal cells induced from human MSCs improves neurological function after stroke without cell fusion.

#### 2.2.2 MSC therapy improves outcomes of stroke by stimulating angiogenesis, neurogenesis, and synapse formation

The mechanisms that underlie the beneficial effects of transplanted MSCs include transdifferentiation into the neural lineage as well as the induction of neurogenesis, angiogenesis, and synapse formation in rodents (Kurozumi et al., 2005; Li et al., 2002; Shen et al., 2006; Wislet‐Gendebien et al., 2005). Transplantation of MSCs protects against cerebral injury and upregulates IL10 expression in Macaca fascicularis (Li et al., 2010), thereby suggesting the activation of endogenous neurotrophins. Angiogenesis that is induced by MSC transplantation promotes endogenous neurogenesis, which may produce functional recovery after cerebral injury in rats with ischemic stroke (Bao et al., 2011). Both histology and MRI reveal that human umbilical MSCs promote recovery after ischemic stroke in rats (Lin et al., 2011). The beneficial effects of MSC therapy are associated with improved revascularization in ischemic injured tissues.

#### 2.2.3 MSC therapy attenuates neuronal death by suppressing activated microglia

During the acute phase of cerebral injury, the expression of neuronal and microglial IL‐6 is elevated in the injured penumbra (Berti et al., 2002; Block, Peters, & Nolden‐Koch, 2000). Direct intrathecal implantation of MSCs results in enhanced neuroprotection. The implantation of MSCs into the injured brain activates resident stem cells niches via an NF kappa B‐mediated increase in IL‐6 production (Walker et al., 2010). Microglia have also been implicated in the pathogenesis of a number of neurodegenerative diseases, such as stroke, Alzheimer's disease, dementia, and multiple sclerosis (Danton & Dietrich, 2003). Microglia can defend against brain damage, but excessive or sustained microglia activation can contribute to apoptotic cell death (Ohmi et al., 2003). Bone marrow MSCs result in the suppression of activated microglia and to a delay of neuronal death (Ohmi et al., 2003; Wei, Fraser, Lu, Hu, & Yu, 2012). Human MSCs also stimulate angiogenesis in focal cerebral injury by increasing expression of α‐tubulin and angiopoietin 1 and 2 (Ma et al., 2013). MSC treatment reduces the expression of inflammatory cytokines in lipopolysaccharide‐activated microglia and subsequently reduces aquaporin‐4 expression and apoptosis of astrocytes after cerebral injury (Tang, Cai, et al. 2014a, Tang, Liu, et al., 2014b). In addition, the survival and function of transplanted MSCs after focal cerebral injury can be enhanced by melatonin pretreatment (Tang et al., 2014b). Both neurological deficit and brain edema and infarct volume are significantly decreased postischemic stroke with MSC treatment via the tail vein (Tang et al., 2014a). MSCs also protect against brain injury in the mouse by stimulating the production of TGF‐β (transforming growth factor), but reduce proinflammatory cytokines (e.g., IL‐1, TNF‐α) (Cheng et al., 2015). Thus, it appears that MSCs improve outcomes of ischemic stroke in animal models by stimulating neurotrophic factors production and endogenous neurogenesis and modulating neuroinflammation.

### 2.3 Stroke patients

Systemic delivery of MSCs has also been shown to be a feasible and safe therapy for treating ischemic stroke patients (Tang et al., 2014b). Long‐term follow‐up data further indicate a contribution of transplanted MSCs to Purkinje neurons in human adult brains (Bang, Lee, Lee, & Lee, 2005; Lee et al., 2010; Weimann, Charlton, Brazelton, Hackman, & Blau, 2003). Both clinical (Bang et al., 2005; Cheng et al., 2015; Tang et al., 2014a) and experimental (Lee et al., 2015) studies demonstrate that the outcomes of ischemic stroke in patients and rodents are greatly improved by MSC therapy. Furthermore, earlier administration of MSCs produces an improved functional recovery, survival rate, stroke recurrences, or adverse effects.

A more recent report has shown that CD4+ CD28‐ T cells (also called CD28 null cells) are increased in the clinical setting of acute ischemic stroke (Tuttolomondo et al., 2015). Among these T cells, CD28 null cells produce high amounts of γ‐interferon and TNF‐α and thus may have a direct pathogenetic role in neuronal damage. It is not known whether the peripheral frequency of CD28 null cells in acute ischemic stroke can be affected by MSC therapy.

## 3 Therapeutic Effects of MSCs in Neural Trauma

### 3.1 Microglial activation as a biomarker for neural trauma

Mechanical injury to the brain or spinal cord results in glutamate excitotoxicity, BBB disruption, ischemia, mitochondria dysfunction, apoptotic and necrotic cell death, and inflammation (Mannix & Whalen, 2012). Secondary injury following TBI (traumatic brain injury) includes microglial activation (Davalos et al., 2005). Microglial activation occurs as early as 72 hr after injury in human TBI patient and persists for years after injury. Activated M1 phenotype microglia causes the overexpression of both proinflammatory cytokines (such as IL‐1β, IL‐6, and TNF‐α) and other neurotoxic products (such as reactive oxygen species [ROS] and reactive nitrogen species [RNS]). M2 phenotype of microglia is able to release neuroprotective substances, including anti‐inflammatory cytokines (IL‐10, IL‐1 receptor antagonist) and neurotrophic factors, including nerve growth factor and transforming growth factor β (TGF‐β) (Chio et al., 2015). Evidence has accumulated that indicates microglial activation as a biomarker for traumatic brain injury (Hernandez‐Ontiveros et al., 2013).

## 4 Therapy with Conditioned Medium from Cultured MSCs Improves Outcomes of Neural Trauma

MSCs transplantations via different routes of administration have been widely studied in different species of SCI (spinal cord injury), and have been proven to produce beneficial effects following SCI (Table 2). The systemic or intraspinal cord administration of MSCs significantly attenuates SCI in rodents (Chopp et al., 2000; Hu et al., 2010; Kao, Chen, Chio, & Lin, 2008; Lu, Jones, & Tuszynski, 2005; Okano et al., 2003; Satake, Lou, & Lenke, 2004), dogs (Penha et al., 2014), rabbits (Moon et al., 2014), monkeys (Deng et al., 2006), or patients (Arien‐Zakay et al., 2014; Cheng et al., 2014; Jiang et al., 2013; Mendonça et al., 2014). MSCs are beneficial in reversing the neurological motor deficits of SCI, even when infused 5 days after injury (Saporta et al., 2003). Human MSCs are observed in the injured areas but not in noninjured areas, of rat spinal cords, and are never seen in corresponding areas of spinal cord of noninjured animals. Immunohistochemical examination reveals that transplanted MSCs survive in the host spinal cord for at least 3 weeks after transplantation but disappear by 5 weeks (Nishio et al., 2006). It is well known that MSC_S_ secrete a variety of molecules that are beneficial in treating SCI (Cantinieaux et al., 2013). Indeed, the systemic administration of conditioned medium (or secretome) from MSCs is shown to improve recovery after SCI in rats (Cantinieaux et al., 2013). In addition, in TBI mice, TBI rats, or TBI patients, intravenous or intrathecal administration of MSCs (Arien‐Zakay et al., 2014; Chen et al., 2014; Lu et al., 2002; Nichols et al., 2013; Wang et al., 2013; Zanier et al., 2011; Zhang et al., 2013, 2015) significantly improves the outcomes of TBI. The systemic injection of the secretome of cultured MSCs also improves the outcomes of TBI in rats (Chang et al., 2013). The transplantation of hypoxia‐preconditioned MSCs improves infracted heart function via the enhanced survival of implanted cells and angiogenesis (Hu et al., 2008). It seems that while MSCs exhibit a prominent multilineage differentiation potential, the MSCs‐derived mediators contribute to cytoprotection, angiogenesis, tissue repair, and alleviation of inflammation during neural injury (Mastri et al., 2014). Cell incorporation into the vessels or neurons is not a prerequisite for the beneficial effects that are exerted by MSCs. MSCs may improve the outcomes of neural injury by modulating multiple mechanisms, such as the secretion of trophic factor and immune function (Kalladka & Muir, 2014).

**Table 2:** Effects of MSCs therapy on spinal cord injury (SCI) or traumatic brain injury (TBI)

Treatment regimens	Main results	References no.
1. SCI rats or SCI mice received intravenous or intraspinal cord injection of MSCs	Reducing spinal cord damage or neurological deficits by stimulating production of both GDNF and VEGF and neurofilament fibers and axonal growth (angiogenesis and neurogenesis).	Chopp et al. (2000), Okano et al. (2003), Satake et al. (2004), Lu et al. (2005), Kao et al. (2008); Hu et al. (2010)
2. SCI rats received intravenous injection of MSCs‐derived secretome	In vitro, secretome obtained from MSCs protects neurons from apoptosis, activates macrophages, and is proangiogenic. In vivo, MSCs secretome improves motor recovery.	Cantinieaux et al. (2013)
3. SCI dogs, rabbits, or monkeys received intraspinal cord injection of MSCs	Reducing spinal cord damage or neurological deficits by stimulating both de novo neurogenesis and production of BDNF	Deng et al., 2006; Penha et al., 2014; Moon et al., 2014
4. SCI patients received intrathecal or intraspinal cord injection of MSCs	Reducing spinal cord damage or neurological deficits	Jiang et al., 2013; Mendonça et al., 2014; Cheng et al., 2014
5. TBI rats or mice received intravenous, intra‐arterial, or intracerebroventricular injection of MSCs	Reducing cerebral damage or neurological deficits by stimulating production of BDNF, NGF, VEGF, and IL‐10, angiogenesis, and neurogenesis.	Lu et al. 2002; Zanier et al., 2011; Zhang et al., 2013, 2015; Nichols et al., 2013; Chen et al., 2014; Arien‐Zakay et al., 2014
6. TBI rats received intravenous injection of MSCs‐derived secretome	Reducing cerebral damage or neurological deficits by secreting bioactive factors, including HGF and VEGF	Chang et al., 2013
7. TBI patients received intrathecal injection of MSCs	Reducing cerebral damage or neurological deficits	Wang et al., 2013

Please see the legends of Table 1 for the explanation of abbreviations.

## 5 Therapeutic Effects of Human MSCs in Experimental Heatstroke

Heatstroke can be induced by severe heat exposure (i.e., classic heatstroke) or strenuous exercise (i.e., exertional heatstroke). Heatstroke syndrome is characterized by critical hyperthermia, which is associated with systemic inflammatory responses that result in multiorgan dysfunction, including delirium, convulsion, or coma (Chen et al., 2013). After the onset of heatstroke, the reduction in blood flow to the brain (or cerebral ischemia) results in hypothalamic neuronal damage, which induces multiple‐organ dysfunction or failure. Heat‐tolerant rats exhibit low levels of both IL‐1β and TNF‐α mRNA in the hypothalamus as well as high corticosterone levels in serum (Hu et al., 2008). In contrast, heat‐intolerant rats present higher hypothalamic levels of both IL‐1β and TNF‐α mRNAs, but lower serum corticosterone level (Michel et al., 2007). Hypothalamic levels of IL‐6, TNF‐α, IL‐1β, and nitrite in the hypothalamus were upregulated by heatstroke (Hsu, Chen, Lin, & Yung, 2014). It has been suggested that the inflammatory response in the acute phase of tissue injury may be related to aggravating tissue injury; however, in the later phase, these inflammatory mediators may contribute to tissue repair (Kadhim, Duchateau, & Sébire, 2008). Cytokines, such as IL‐6 and TNF‐α, may be essential at physiological levels for the maintenance of the endogenous neurogenesis in the brain (Bowen et al., 2011). Neither lack nor excess of IL‐6 or TNF‐α is beneficial for homeostasis of the inflammatory mechanisms.

### 5.1 Resuscitation from experimental heatstroke by transplantation of human umbilical cord blood cells (HUCBC) or HUCBC‐derived CD34+ cells

The plasma levels of inflammatory cytokines, such as IL‐6 and TNF‐α, are elevated in humans (Bouchama, Al‐Sedairy, Siddiqui, Shail, & Rezeig, 1993; Chang, 1993), rats, and rabbits (Lin, Kao, Su, & Hsu, 1994; Lin, Liu, & Yang, 1997) and mice with heatstroke (Tseng, Chen, Lin, & Lin, 2014). HUCBCs improve outcomes of heatstroke by reducing circulatory shock, cerebral injury (Chen, Chang, Tsai, Huang, & Lin, 2005; Chen et al., 2006), and systemic inflammation (Chen et al., 2006; Liu et al., 2009). The administration of HUCBC increases the serum levels of IL‐10 and decreases the levels of TNF‐α during heatstroke (Liu et al., 2009; Tseng et al., 2014).

It has been estimated that approximately 2% of HUCBC are positive for CD34 expression (Bender et al., 1991). CD34+ cells transplantation also attenuates the outcomes of heatstroke by reducing TNF‐α production in serum, stimulating IL‐10 production in serum, and stimulating production of GDNF in brain (Chen et al., 2007; Hwang et al., 2008).

### 5.2 The potential use of granulocyte‐colony stimulating factor (G‐CSF) as a prophylactic agent for heatstroke

G‐CSF is a polypeptide that promotes the mobilization of stem cells into peripheral blood (Lu & Xiao, 2006) and results in a reduction in mortality, infarct volume, and neurological deficits after cerebral ischemia in heatstroke rats (Lu & Xiao, 2006). Preconditioning with G‐CSF attenuates heatstroke‐induced hypothalamic apoptosis and neuronal damage by stimulating GDNF and VEGF overproduction in hypothalamus, thereby reducing levels of TNF‐α, increasing levels of IL‐10, and stimulating the expression of endothelial progenitor cells in the serum of rats (Yung et al., 2011).

### 5.3 Transplantation of human dental pulp‐derived stem cells protects against heatstroke

Human dental pulp‐derived stem cells are self‐renewing stem cells that reside within the perivascular niche of the dental pulp (Gronythos et al., 2002). They enhance recovery of focal cerebral injury in rats (Inoue et al., 2013). Human dental pulp‐derived stem cells are also shown to attenuate ischemia and oxidative damage to the hypothalamus and the overproduction of systemic response syndrome molecules, including TNF‐α and ICAM‐1, in the peripheral blood stream in heatstroke mice (Tseng, Chen, Lin, & Lin, 2015). When considering the data presented herein, it appears that human MSCs may improve outcomes of heatstroke by reducing the overproduction of systemic response syndrome molecules as well as multiple‐organ dysfunction or failure (Table 3).

**Table 3:** Effects of mesenchymal stem cells (MSCS) therapy on heatstroke‐induced cerebral ischemic damage

Treatment regimens	Main results	References no.
1. Heatstroke rats received intravenous or intracerebroventricular injection of HUCBCs	MSCs attenuate cerebral ischemic damage by reducing overproduction of TNF‐α, IL‐1β, and IL‐6, but stimulating production of IL‐10.	Chen et al. (2005) Chen et al. (2006) Liu et al. (2009)
2. Heatstroke rats received intravenous injection of HICBC‐derived CD34+ cells	MSCs reduce cerebral ischemic damage by reducing overproduction of both TNF‐α and ICAM‐1, but stimulating production of IL‐10.	Hwang et al. (2008) Chen et al. (2007) Tseng et al. (2014)
3. Heatstroke rats received subcutaneous injection of granulocyte‐colony stimulating factor	The factor attenuates cerebral ischemic damage by reducing overproduction of both TNF‐α and ICAM‐1, but stimulating production of IL‐10, EPSs, GDNF, and VEGF.	Yung et al. (2011)
4. Heatstroke mice received intravenous injection of human dental pulp‐derived stem cells or HUCBCs	MSCs attenuate cerebral ischemic damage by reducing overproduction of TNF‐α, intercellular adhesion molecule 1, and oxidative damage markers, but promoting both hypothalamo–pituitary–adrenocortical axis activity and IL‐10 production.	Tseng et al. (2014) Tseng et al. (2015)

HUCBCs, human umbilical cord blood cells.

Please see the legends of Table 1 for the explanation of abbreviations.

**Figure 1:**
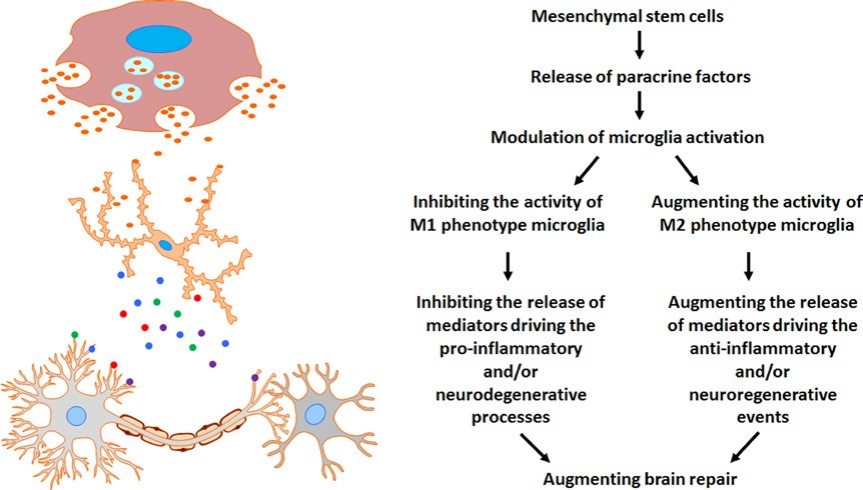
The mechanisms of MSC therapy‐related neuroprotection. Microglia are the first source of the inflammatory cascade in brain injury. Microglia are activated rapidly in response to central nervous system injury and produce proinflammatory cytokines, growth factors, reactive oxygen species, nitric oxide, and glutamate (Block & Hong, 2005; Jin et al., 2010; Stolp & Dziegielewska, 2009). The proinflammatory M1 phenotype of microglia is associated with tissue destruction, whereas the anti‐inflammatory M2 phenotype of microglia facilitates repair and regeneration. During brain injury, the activity of M1 phenotype microglia and M2 phenotype microglia is augmented and inhibited, respectively. In contrast, MSCs might improve outcomes of brain injury by inhibiting the activity of M1 phenotype microglia and augmenting the activity of M2 phenotype microglia

Therefore, it can be concluded that some spontaneous but not extensive recovery (or increased endogenous neurogenesis) is typical following brain injury caused by stroke, neural trauma, and heatstroke. Exogenous cell therapy is promising as a means of augmenting brain repair by modulating microglial activation as depicted in Fig. 1. As mentioned in the foremost section, the proinflammatory M1 phenotype of microglia is associated with tissue destruction, whereas the anti‐inflammatory M2 phenotype of microglia facilitates repair and regeneration. Therefore, MSC therapy may improve outcomes of ischemic stroke, neural trauma, and heatstroke by inhibiting the activity of M1 phenotype of microglia but augmenting the activity of M2 phenotype of microglia.

## 6 Conclusions

### 6.1 The targeting of microglial activation in clinical trials as a rational design of NSC therapy in the future

During ischemic stroke, after brain trauma, and during heatstroke, the intrinsic inflammatory mechanisms of the brain as well as those of the peripheral blood stream are mediated by the release of pro‐ and anti‐inflammatory cytokines and chemokines. According to a more recent review (Chio et al., 2015; Tuttolomondo, Pecoraro, & Pinto, 2014), microglia are the first source of the inflammatory cascade during brain ischemia and after brain trauma. Additionally, an important mediator of this inflammatory event is TNF‐α. Etanercept, a TNF‐α antagonist, which has been used therapeutically in animal models of ischemic stroke and neural damage (Kinnaird et al., 2004). In addition, various drugs or strategies have improved the outcomes of experimental heatstroke by reducing the overproduction of these proinflammatory cytokines in both the brain and the peripheral blood stream (Chen et al., 2013). Although some spontaneous recovery (due to endogenous neurogenesis) in humans is thought to contribute to repair, exogenous MSC therapy is promising as a means of augmenting brain repair. MSCs, when administered systemically, are observed in the injured brain areas but not in noninjured brain areas and are never seen in corresponding brain areas of noninjured animals (Nishio et al., 2006). Nevertheless, paracrine signaling, rather than cell incorporation into vessels or neurons, is a prerequisite for the beneficial effects that are exerted by MSCs (Kinnaird et al., 2004). The multiple mechanisms of action of MSCs include enhanced angiogenesis and neurogenesis (by the secretion of trophic factors), immunomodulation, and anti‐inflammatory effects (Kalladka & Muir, 2014). Cytokines, including TNF‐α, IL‐1, and IL‐6, are greatly produced by microglia in the brain after experimental stroke (Clausen et al., 2005, 2008; Hill et al., 1999; Lambertsen et al., 2005). Appropriate baseline levels of TNF‐α or IL‐6 are necessary and essential for neurogenesis or host defense, whereas higher levels of TNF‐α or IL‐6 are detrimental to neurogenesis or host defense (please see the Introduction). The appropriate level of microglial activation is necessary and crucial for normal neurogenesis and host defense, whereas microglial overactivation results in deleterious and neurotoxic consequences (please see the Introduction section). The exogenous administration of MSCs may promote tissue repair by stimulating trophic factor release and endogenous neurogenesis (Chamberlain, Fox, Ashton, & Middleton, 2007; Chen, Tredget, Wu, & Wu, 2008; Phinney & Prockop, 2007). The expression of prosurvival and proangiogenic markers in MSCs can be enhanced by hypoxic preconditioning (Chacko et al., 2010). This review offers a testable platform for the targeting of microglial activation in clinical trials that are based upon rational design of MSC therapy in the future.

It is most probable that the central concern that is considered in this review is that MSCs may exert their neuroprotective effects mainly by modulating the production of both cytokines and neurotrophic factors. In addition, immunosuppression of allogenic MSC transplantation after neural injury improves graft survival and beneficial outcomes in rats (Torres‐Espín, Redondo‐Castro, Hernandez, & Navarro, 2015). Conversely, the intravenous, intranasal, or intracerebral administration of MSCs is found to be beneficial in treating neurological damage. To produce a similar beneficial effect, the intravenous route injection needs a lesser dosage than does the intracerebral route injection, suggesting the central action of MSCs. Additionally, compared to MSCs therapy adopted postinjury, pretreatment regimens of MSCs has significantly better beneficial effects. However, expansion of MSCs without fetal bovine serum is a big problem during different pretreatment regimens and preparation of MSCs. Nevertheless, because substances that are administered via the peripheral blood stream are able to reach multiple organs (including brain tissues) during diseased conditions, intravenous route injection is the most practical approach for cell therapy in general.

### 6.2 MSCs that are derived from the placenta may be the most practical for use in the treatment of brain ischemic injury

To our knowledge, the in vitro characteristics and in vivo potency of placenta‐derived MSCs have not been well explored or investigated thoroughly. In addition, future studies are warranted for the clinical application of placenta‐derived MSCs in stroke, TBI, heatstroke, and other neurodegenerative diseases. In our opinion, MSCs fulfill the criteria that have been set by the International Society of Cellular Therapy (as described in the Introduction section). Therefore, in comparison with other types of stem cells, MSCs are the most practical to use in the case of brain ischemic injury.

## Funding Information

Ching‐Ping Chang and Mao‐Tsun Lin have been supported by Ministry of Science and Technology of the Republic of China, Taipei, Taiwan (no. MOST 104‐2314‐B‐218‐001‐MY3; CMFHT 10401).

## Conflict of Interest

The authors confirm that this article content has no conflict of interest.
